# Amelioration of Colitis by a Gut Bacterial Consortium Producing Anti-Inflammatory Secondary Bile Acids

**DOI:** 10.1128/spectrum.03330-22

**Published:** 2023-03-21

**Authors:** Chunhua Zhou, Ying Wang, Cun Li, Zhiyong Xie, Lei Dai

**Affiliations:** a School of Pharmaceutical Sciences (Shenzhen), Shenzhen Campus, Sun Yat-sen University, Shenzhen, China; b CAS Key Laboratory of Quantitative Engineering Biology, Shenzhen Institute of Synthetic Biology, Shenzhen Institute of Advanced Technology, Shenzhen, China; Shenzhen Bay Laboratory

**Keywords:** gut microbiome, colitis, bacterial consortium, secondary bile acids, targeted metabolomics, metabolomics

## Abstract

The Integrative Human Microbiome Project and other cohort studies have indicated that inflammatory bowel disease is accompanied by dysbiosis of gut microbiota, decreased production of secondary bile acids, and increased levels of primary bile acids. Secondary bile acids, such as ursodeoxycholic acid (UDCA) and lithocholic acid (LCA), have been reported to be anti-inflammatory, yet it remains to be studied whether introducing selected bacteria strains to restore bile acid metabolism of the gut microbiome can alleviate intestinal inflammation. In this study, we screened human gut bacterial strains for bile acid metabolism and designed a consortium of three species, including *Clostridium* AP sp000509125, Bacteroides ovatus, and Eubacterium limosum, and named it BAC (bile acid consortium). We showed that the three-strain gut bacterial consortium BAC is capable of converting conjugated primary bile acids taurochenodeoxycholic acid and glycochenodeoxycholic acid to secondary bile acids UDCA and LCA *in vitro*. Oral gavage treatment with BAC in mice resulted in protective effects against dextran sulfate sodium (DSS)-induced colitis, including reduced weight loss and increased colon length. Furthermore, BAC treatment increased the fecal level of bile acids, including UDCA and LCA. BAC treatment enhanced intestinal barrier function, which may be attributed to the increased activation of the bile acid receptor TGR5 by secondary bile acids. Finally, we examined the remodeling of gut microbiota by BAC treatment. Taken together, the three-strain gut bacterial consortium BAC restored the dysregulated bile acid metabolism and alleviated DSS-induced colitis. Our study provides a proof-of-concept demonstration that a rationally designed bacterial consortium can reshape the metabolism of the gut microbiome to treat diseases.

**IMPORTANCE** Secondary bile acids have been reported to be anti-inflammatory, yet it remains to be studied whether introducing selected bacteria strains to restore bile acid metabolism of the gut microbiome can alleviate intestinal inflammation. To address this gap, we designed a consortium of human gut bacterial strains based on their metabolic capacity to produce secondary bile acids UDCA and LCA, and we evaluated the efficacy of single bacterial strains and the bacterial consortium in treating the murine colitis model. We found that oral gavage of the bacterial consortium to mice restored secondary bile acid metabolism to increase levels of UDCA and LCA, which induced the activation of TGR5 to improve gut-barrier integrity and reduced the inflammation in murine colitis. Overall, our study demonstrates that rationally designed bacterial consortia can reshape the metabolism of the gut microbiome and provides novel insights into the application of live biotherapeutics for treating IBD.

## INTRODUCTION

Inflammatory bowel disease (IBD) is a chronic systemic inflammatory condition (1, 2); the two main clinical phenotypes of IBD are Crohn’s disease and ulcerative colitis (3). IBD affects 6.8 million people globally (4) and causes huge medical expenses. Although the mechanism of IBD is unclear, numerous factors might contribute to IBD pathogenesis, including host genetics, immune dysregulation, and gut microbiome (5, 6).

Multiple studies have found that dysbiosis of the gut microbiome is closely related to IBD (7, 8). A critical function of the gut microbiome in regulating host health is via derived metabolites (9–11). Hundreds of gut microbiota-derived metabolites have been identified by targeted and untargeted metabolomics, including bile acids, short-chain fatty acids, branched-chain amino acids, trimethylamine *N*-oxide, tryptophan, and indole derivatives (12–15). In particular, cohort studies, including the Integrative Human Microbiome Project (iHMP), have revealed that the pathogenesis of IBD is accompanied by a decrease in secondary bile acids (7). Supplementation of secondary bile acids has been found to reduce inflammation in both acute and chronic murine colitis models (16).

Primary bile acids are synthesized from cholesterol in the liver, reach the intestine mainly through postprandial release into the gut lumen, and are reabsorbed through enterohepatic circulation. In the lower part of the small intestine and large intestine, some primary bile acids are metabolized to secondary bile acids by the gut microbiome (17, 18). Primary bile acids are conjugated via an amide bond to glycine or taurine to increase solubility. The deconjugation of primary bile acid in the colon depends on the enzymatic activity of bile salt hydrolases (BSH) present in some commensal bacteria (19). The deconjugated bile acids then undergo biotransformation mediated by 7α-dehydroxylation enzymes of gut microbiota to generate secondary bile acids (e.g., lithocholic acid [LCA] and deoxycholic acid [DCA]) (20). In addition, primary bile acids may be isomerized by a hydroxysteriod dehydrogenase (HSDH), such as 3α/βHSDH or 7α/βHSDH (21, 22).

The restoration of secondary bile acid production via the gut microbiome provides a promising target for treating IBD. There have been attempts to treat IBD patients with fecal bacteria transplantation, but enrichment-based approaches have several disadvantages (6), including transferable antibiotic resistance functions or undesirable strains or functions associated with safety risks, including virulence factors. In contrast, an alternative approach is to rationally select bacteria strains that restore the disrupted function of the dysbiotic gut microbiome of IBD patients (23).

In this study, we designed a consortium of human gut bacterial strains based on their metabolic capacity to produce secondary bile acids ursodeoxycholic acid (UDCA) and LCA, which have been shown to be depleted in IBD patients. Our bile acid consortium (referred to as BAC here) consists of 3 bacterial species isolated from healthy human donors, including *Clostridium* AP sp000509125, Bacteroides ovatus, and Eubacterium limosum. We then applied the BAC to the dextran sulfate sodium (DSS)-induced colitis mouse model. We showed that the BAC elevated the levels of secondary bile acids *in vivo*, ameliorated colitis symptoms, and reshaped the gut microbiota. Our study provides novel insights into the design of synthetic bacterial consortium for preventing and treating IBD.

## RESULTS

### Screening human gut bacterial strains for bile acid metabolism.

The iHMP study found that decreases of secondary bile acids were prevalent in IBD patients. An independent cohort study of ulcerative colitis patients also showed that the level of the primary bile acid chenodeoxycholic acid (CDCA) was higher in patients, while the secondary bile acid LCA was lower (16). Moreover, it has been shown that secondary bile acids LCA and UDCA can alleviate colitis (24). Consistent with previous findings, we found that LCA and UDCA mitigated the symptoms of colitis in the DSS mouse model, confirming their anti-inflammatory effects (see Fig. S1 in the supplemental material). This provided a molecular basis for our screening of gut bacterial strains involved in bile acid metabolism.

Previous studies have shown that the gut microbiome plays a critical role in bile acid metabolism (3, 25). BSH enzymes of gut bacteria hydrolyze conjugated bile acids to unconjugated bile acids (26). Cholic acid (CA) and CDCA are then converted into deoxycholic acid (DCA) and LCA by 7α-dehydroxylation mediated by enzymes of the bile acid-inducible (bai) operon (20, 27, 28). In addition, CDCA can be converted to UDCA by 7-hydroxyl epimerization mediated by 7α/βHSDH enzymes (29, 30). We therefore aimed to identify gut bacterial strains involved in bile acid metabolism to design a synthetic consortium to convert conjugated bile acids (taurochenodeoxycholic acid [TCDCA] and glycochenodeoxycholic acid [GCDCA]) to secondary bile acids (UDCA and LCA) to prevent and treat colitis.

**FIG 1:**
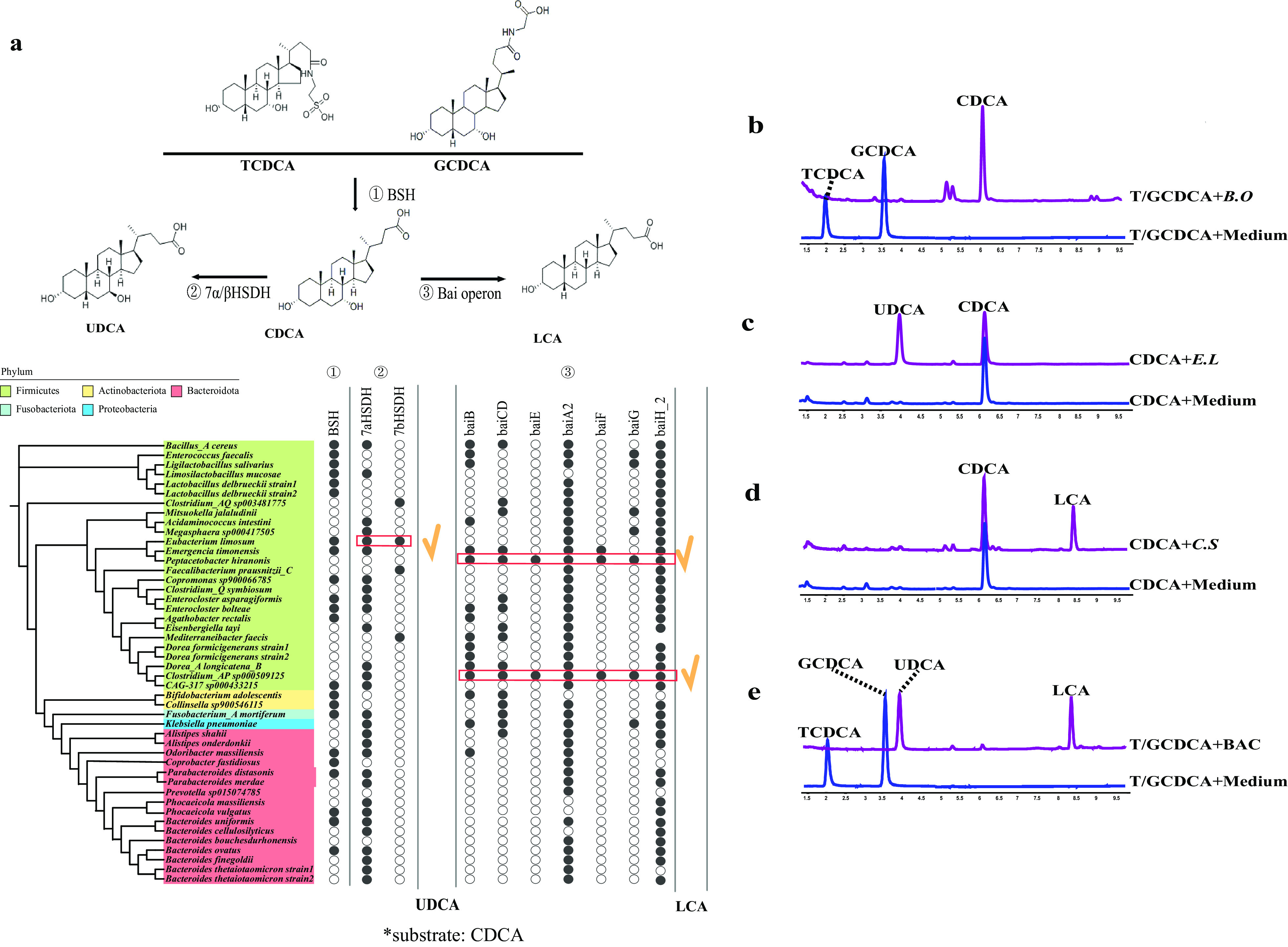
Bioinformatics analysis and experimental validation of bile acid metabolism of human gut bacterial strains. (a) Homology-based search in the whole-genome sequence of human gut bacterial strains identified genes related to bile acid metabolism, including BSH, 7α/βHSDH, and the bai operon. Red rectangles, *E. limosum* with 7α/βHSDH, *Clostridium* AP sp000509125, and *Peptacetobacter* with the complete bai operon. (b) *B. ovatus* converts TCDCA and GCDCA into CDCA. (c) *E. limosum* converts CDCA into UDCA. (d) *Clostridium* AP sp000509125 converts CDCA into LCA. (e) The three-strain consortium BAC (*B. ovatus*, *E. limosum*, and *Clostridium* AP sp000509125) converts TCDCA and GCDCA into UDCA and LCA. In our experimental validation, bacterial strains were incubated with TCDCA and GCDCA or CDCA for 60 h and then subjected to LC/MS description. G, glyco-; T, tauro-; UDCA, ursodeoxycholic acid; LCA, lithocholic acid; CDCA, chenodeoxycholic acid; *B.O*, Bacteroides ovatus; *E.L*, Eubacterium limosum; *C.S*, *Clostridium* AP sp000509125.

Combining bioinformatics analysis and targeted metabolomics (see Materials and Methods), we focused on screening the gut bacterial strains that can complete the conversion of conjugated bile acids TCDCA and GCDCA to secondary bile acids LCA and UDCA via the combined enzymatic activities of BSH, 7α/βHSDH, and the bai operon (Fig. 1a). We included 46 human gut bacterial strains in the screening, which were isolated from fecal samples of healthy volunteers and covered 5 phyla. The 46 strains used in this study were selected from an in-house collection of ~300 gut bacterial species isolated from the fecal samples of healthy donors. Using the whole-genome sequencing data of these in-house gut bacterial species, we identified 46 strains with at least one enzyme related to bile acid metabolism (Fig. 1a) and included them for *in vitro* screening.

Previous studies suggested that the gut commensal *Bacteroides* can hydrolyze TCDCA to taurine and CDCA (26, 31–33). We identified the presence of BSH in the genome of Bacteroides ovatus and validated its function *in vitro* (Fig. 1b). Moreover, by adding CDCA in the growth medium, we screened for human gut bacterial strains that can convert CDCA to secondary bile acids UDCA and LCA *in vitro*. The targeted metabolomics results showed that, among the 46 strains, only Eubacterium limosum converted CDCA to UDCA (Fig. 1c). Finally, we found that *Clostridium* AP sp000509125 converted CDCA to LCA (Fig. 1d). Our metabolic assays were consistent with the genomic analysis of 46 strains (Fig. 1a), which indicated that 7-a/βHSDH was only present in Eubacterium limosum, and homologous genes to the bai operon were only present in the genomes of *Clostridium* AP sp000509125 and *Peptacetobacter* (34–36).

Therefore, we chose to include Bacteroides ovatus, Eubacterium limosum, and *Clostridium* AP sp000509125 in the BAC, which included BSH, 7α/βHSDH enzymes, and the bai operon. We cocultured three bacterial strains *in vitro* and found that the three-strain BAC could indeed convert conjugated bile acids TCDCA and GCDCA to the anti-inflammatory secondary bile acids UDCA and LCA (Fig. 1e).

### BAC treatment alleviated symptoms of the DSS-induced colitis and elevated the fecal concentration of bile acids.

**FIG 2:**
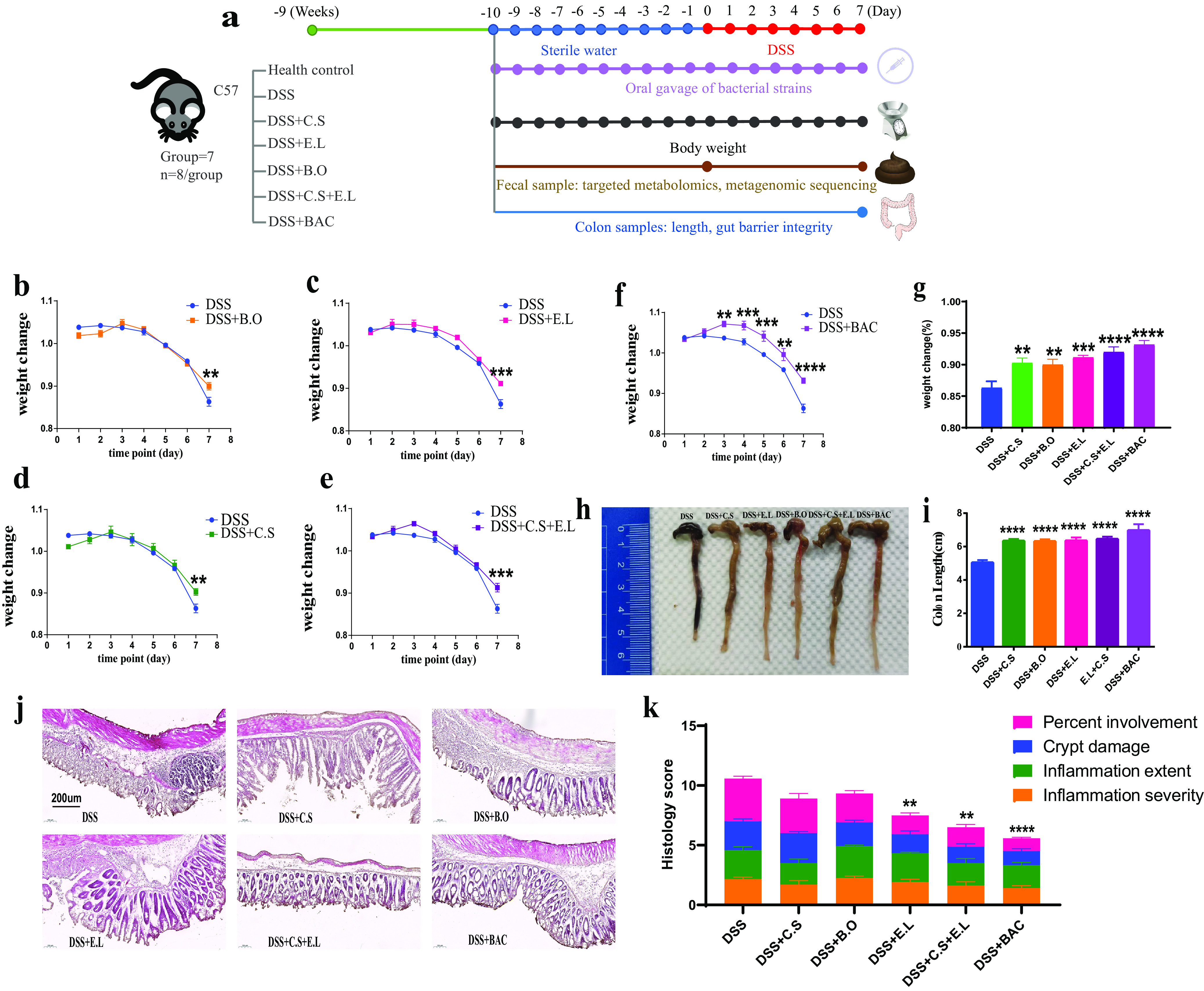
Three-strain consortium BAC ameliorated DSS-induced colitis. (a) Design of animal experiments. C57BL/6 mice were treated with 2% DSS (wt/vol) for 7 days before sacrifice. Bacterial strains (single strain or in consortium) were orally administered on a daily basis. For the DSS group, BHI medium was orally administered as a control. (b to f) Oral administration of BAC ameliorated weight loss during DSS treatment. (g) Animal weights on the last day of DSS treatment. (h) Representative colon images of sacrificed mice. (i) Oral administration of BAC restored colon length. (j and k) Colon inflammation and histopathology score. B.O, Bacteroides ovatus; *E.L*, Eubacterium limosum; *C.S*, *Clostridium* AP sp000509125. The mean and standard error of the mean (SEM) of each group are shown (*n* = 5 to 8 mice). Each treatment group was compared with the DSS group using a one-way ANOVA (g, i, and k) or two-way ANOVA (b, c, d, e, and f) followed by the Bonferroni *post hoc* test. *, *P* < 0.05; **, *P* < 0.01; ***, *P* < 0.001; ****, *P* < 0.0001.

To investigate the protective effects of the BAC, we used individual strains and the combination of multiple strains to treat the DSS-induced colitis in mice. The treatment group was administered a single strain or a consortium (total, 1 × 10^9^ CFU) on a daily basis for 10 days, followed by 2% DSS administration in drinking water for 7 days (Fig. 2a). In comparison to the DSS control group, we found that mice in all treatment groups had reduced symptoms of DSS-induced colitis (Fig. 2b to k). Moreover, the BAC treatment group showed the most significant protective effects, as illustrated by reduced weight loss, increased colon length, and reduced colon histopathology compared with the DSS control group (Fig. 2g to k).

**FIG 3:**
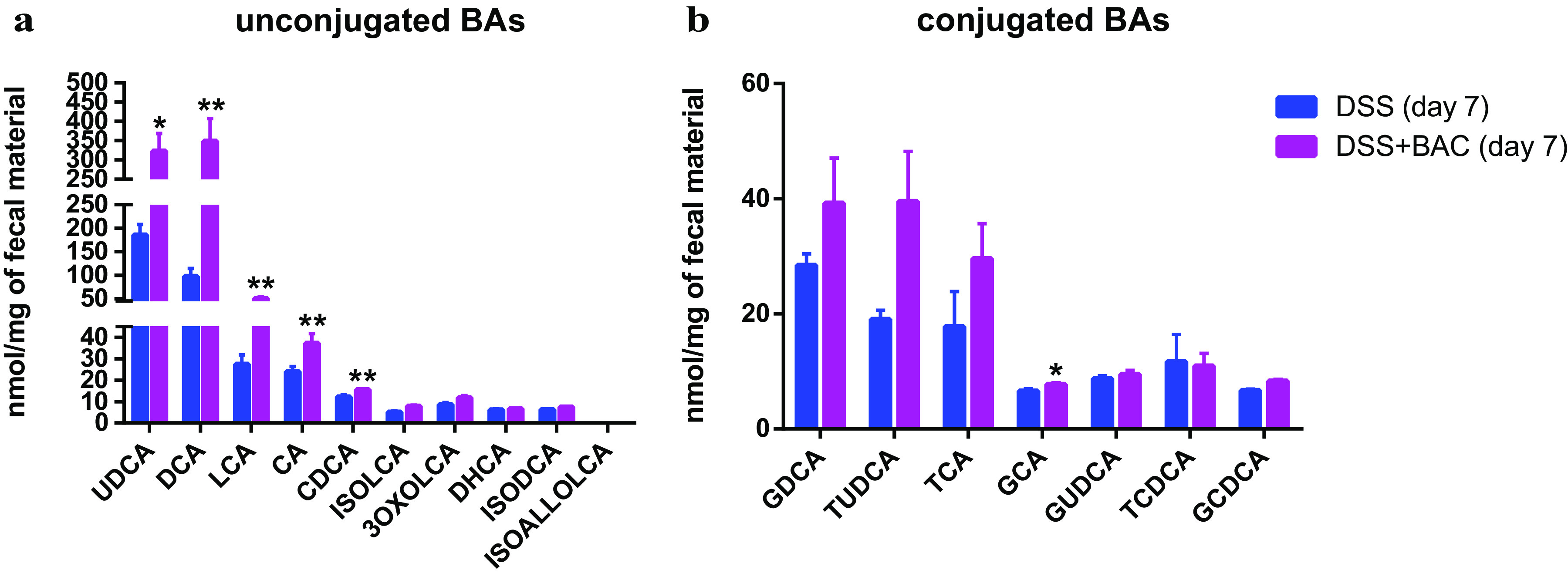
Treatment of three-strain consortium BAC increased secondary bile acids in mouse fecal samples. Targeted metabolomic analysis of unconjugated bile acids (a) and conjugated bile acids (b). The mean and SEM of each group are shown (*n* = 8 mice). Two-tailed Student's *t* test (or Mann-Whitney test) was used. *, *P* < 0.05; **, *P* < 0.01.

To evaluate the effects of the BAC on bile acid metabolism of the gut microbiome, we measured the levels of 17 bile acids in mouse fecal samples, including both conjugated and free bile acids, by targeted metabolomics. Compared to the DSS control group, BAC treatment substantially altered the fecal levels of bile acids (Fig. 3). In particular, we observed a significant increase in secondary bile acids UDCA, DCA, and LCA. Thus, consistent with the *in vitro* metabolic assays, we found that the 3-strain BAC elevated bile acid production *in vivo*. Since the levels of secondary bile acids were reported to be lower in DSS-induced colitis mice and IBD patients (7, 16) than in healthy hosts, the restoration of bile acid metabolism by BAC treatment was consistent with the observed amelioration of colitis symptoms.

### BAC treatment promotes gut barrier integrity.

**FIG 4:**
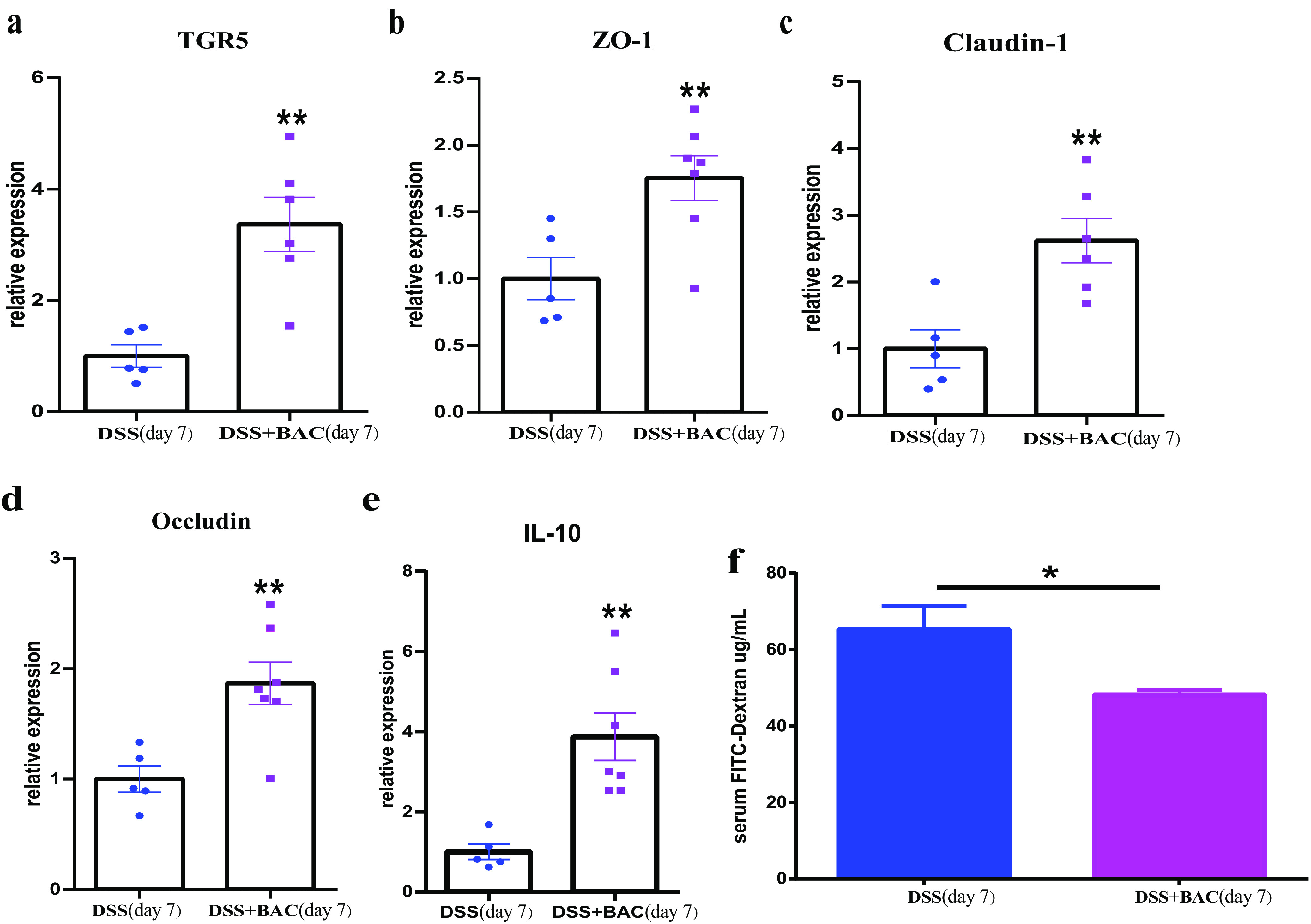
Three-strain consortium BAC restored gut barrier integrity in DSS-induced colitis. (a-e) Gene expression of TGR5 (a membrane receptor for bile acids), tight junction proteins ZO-1, Claudin-1, Occludin, and IL-10. Gene expression levels in colon tissue were assayed by qPCR. (f) FITC-dextran assay indicated that BAC treatment decreased intestinal paracellular permeability (i.e., restoration of gut barrier integrity). The mean and SEM of each group are shown (DSS group: *n* = 5 mice; DSS+BAC group: *n* = 7 mice). Two-tailed Student's *t* test (or Mann-Whitney test), *, *P* < 0.05, **, *P* < 0.01.

TGR5 is a membrane receptor for bile acids expressed in the distal ileum and colon epithelium (37). TGR5 activation has been reported to promote intestinal renewal, increase intestinal barrier function, and increase the level of anti-inflammatory cytokines such as interleukin-10 (IL-10) in response to bile acid signals (38, 39). We tested the effects of the BAC treatment on TGR5 signaling. Quantitative PCR (qPCR) results with colon tissues showed that the expression of TGR5 was increased by BAC treatment compared to the DSS control group (Fig. 4a). Moreover, we found that BAC treatment increased the expression of tight junction proteins *ZO-1*, *Claudin-1*, and *Occludin* and the expression of the anti-inflammatory cytokine IL-10 (Fig. 4b to e).

To test whether BAC treatment promoted gut barrier integrity, we assayed the intestinal paracellular permeability using fluorescein isothiocyanate-dextran (FITC-dextran). Mice that received the BAC treatment showed decreased intestinal paracellular permeability compared to the DSS control group (Fig. 4g), consistent with the increased expression levels of tight junction proteins. Taken together, our results indicated that BAC treatment protected against the damage of the intestinal barrier by DSS-induced colitis.

### Effects of BAC treatment on the gut microbiome.

Finally, we investigated the effects of BAC treatment on the gut microbiome by metagenomic sequencing. We collected mouse fecal samples at the beginning of the experiment (i.e., baseline) and on the last day of DSS treatment (Fig. 2a). As expected, in comparison to the healthy mice (i.e., control group), we observed substantial shifts in the composition of the murine gut microbiome after DSS treatment (i.e., DSS group), such as a decrease in *Muribaculum*.

**FIG 5:**
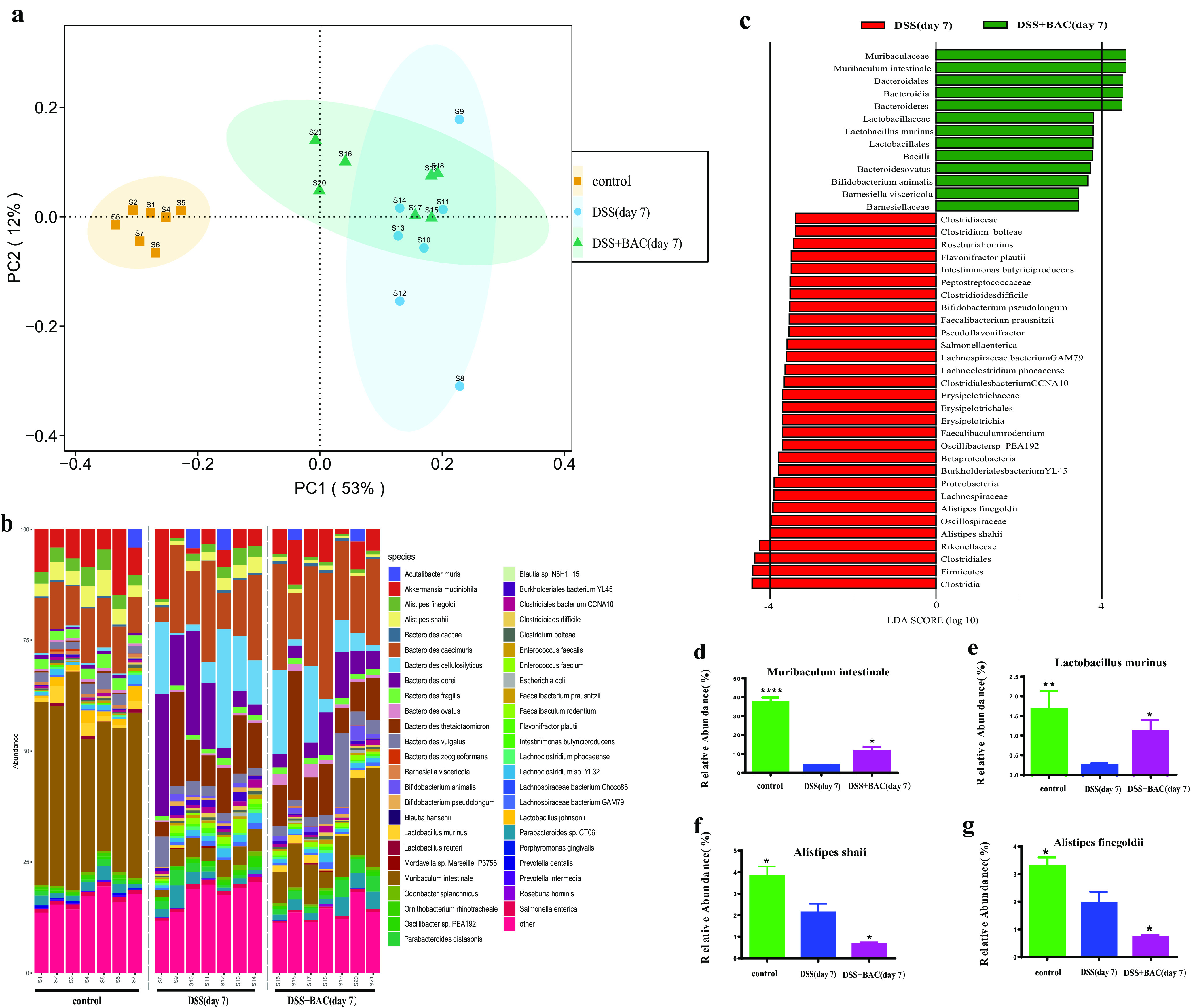
Three-strain consortium BAC reshaped gut microbiota in DSS-induced colitis mice. (a) PCoA of the murine gut microbiota of three different groups. (b) Compositional profile of murine gut microbiota at the species level. (c) Bacterial taxa identified as differentially abundant between the untreated group (DSS) and the BAC treatment group (DSS + BAC) by LEfSe. Green indicates bacterial taxa whose abundance was higher in the DSS + BAC group; red indicates otherwise. (d to g) The relative abundance of bacterial species of each group was compared with DSS group (*n* = 7), using a one-way ANOVA followed by the Bonferroni *post hoc* test. *, *P* < 0.05; **, *P* < 0.01; ***, *P* < 0.001; ****, *P* < 0.0001.

Compared to the DSS group, we found that BAC treatment (i.e., DSS + BAC group) altered the murine gut microbiome at both the species level (Fig. 5a and b) and the genus level (Fig. S2). In particular, the relative abundance of Muribaculum intestinale and Lactobacillus murinus increased in the BAC treatment group (Fig. 5c to g); in contrast, the relative abundance of Alistipes shahii and Alistipes finegoldii decreased. *Muribaculum instestinale* is the dominant species in the murine gut microbiome (40), and its abundance was partially restored after BAC treatment. Lactobacillus murinus was previously found to reduce intestinal permeability and attenuate systemic inflammation (41).

Based on metagenomic sequencing of mouse fecal samples, we found that the relative abundance of Bacteroides ovatus and Eubacterium limosum in the BAC-treated mice was significantly higher than in the untreated mice on day 0 (i.e., after 10 days of oral gavage). On day 7 (i.e., at the end of DSS-induced colitis), we found that the relative abundance of Bacteroides ovatus in BAC-treated mice was still higher than that in untreated mice. Interestingly, the relative abundance of Eubacterium limosum in untreated mice increased during colitis and reached a level similar to the BAC-treated group (Fig. S4). At both time points (day 0 and day 7), the relative abundance of *Clostridium* AP sp000509125 was below the detection limit.

## DISCUSSION

In this study, we combined genomic analysis and *in vitro* metabolic assays to design a bacterial consortium (BAC) that can convert TCDCA and GCDCA to UDCA and LCA. The BAC promoted the production of secondary bile acids *in vivo* and ameliorated inflammation in DSS-induced murine colitis.

Bile acid receptors include farnesoid X receptor, the pregnane X receptor, the vitamin D receptor, and the G-protein-coupled receptor TGR5 (26). Secondary bile acids LCA and DCA are strong activators of TGR5 (17). We found that BAC treatment increased the production of secondary bile acids as well as colon expression of TGR5 in DSS-treated mice. These bile acid receptors may alleviate the symptoms of inflammation through inhibition of NLRP3 inflammasome (42) or by promoting the polarization of macrophages toward an anti-inflammatory phenotype (43).

Moreover, we found that BAC treatment led to increases in the expression of junction proteins *ZO-1* and *Claudin-1*, indicating that BAC treatment conferred protection against gut barrier damage in DSS-induced colitis. A Luminex multiplex immunoassay of serum samples showed that the BAC treatment had profound effects on the chemokine and cytokine profiles associated with inflammation, including decreases of proinflammatory cytokines tumor necrosis factor alpha, IL-17A, and IL-6 (Fig. S3). Therefore, we propose the potential therapeutic mechanism of BAC treatment as the following: BAC restored secondary bile acid metabolism to increase levels of UDCA and LCA, which induced the activation of TGR5 to improve gut-barrier integrity and reduce inflammation in murine colitis.

There are several caveats of this study. First, while the relative abundance of Bacteroides ovatus significantly increased in the treatment group on day 7, we did not find an increase in the relative abundance of Eubacterium limosum in metagenomic analysis of fecal samples. The relative abundance of *Clostridium* AP sp000509125 was below the detection limit of metagenomic sequencing, indicating that this strain may be unable to colonize the murine gut. In future studies, it would be desirable to screen for strains that can stably colonize in the gut, or to express the relevant enzymes in engineered commensal gut bacteria. Second, in our experiments the inoculation of BAC preceded the DSS-induced colitis; thus, the effect was mostly prophylactic rather than rescue. Potential application of BAC as live biotherapeutics in treating human IBD may be limited to patients at an early stage of the disease. Third, *Eubacteriaceae* and *Bacteroidaceae* have been reported to produce other metabolites with anti-inflammatory potential (e.g., short-chain fatty acids), which were not investigated in our study.

In conclusion, the three-strain gut BAC restored the dysregulated bile acid metabolism and alleviated DSS-induced colitis. Our study provides a proof-of-concept demonstration that a rationally designed bacterial consortium can reshape the metabolism of the gut microbiome to treat diseases.

## MATERIALS AND METHODS

### Bacterial culture.

Bacterial strains were cultured in an anaerobic chamber (Coy; 85% N_2_, 10% H_2_, and 5% CO_2_) at 37°C. Bacteria strains were cultured in brain heart infusion (BHI) media supplemented with 5 mg/liter hemin, 0.5 mg/liter vitamin K_3_, and 1 g/L cysteine-HCl. The 46 strains used in this study were selected from an in-house collection of ~300 gut bacterial species isolated from the fecal samples of healthy donors (unpublished data). Fecal samples of healthy human donors were collected and immediately transferred to an anaerobic chamber, homogenized in phosphate-buffered saline (PBS) supplemented with 0.1% cysteine, and then diluted and spread on agar plates containing growth medium. Plates were incubated anaerobically at 37°C for 2 to 3 days. Single colonies were picked, streaked onto a new plate, and incubated anaerobically at 37°C for another 2 to 3 days. The purification steps were repeated several times. All purified strains were stored at −80°C in glycerol suspension (25% [vol/vol]) containing 0.1% cysteine.

### Bioinformatics analysis.

The whole-genome sequence of 46 human gut bacterial strains was queried by Blastp to identify homologous proteins (>30% identity and >70% coverage to the query sequence) of the bai operon, the BSH gene, and the 7α/7βHSDH gene. The following accession numbers were utilized: bai operon, GenBank accession number U57489.2; 7βHSDH, UniProtKB/Swiss-Prot accession number G9FRD6.1; 7αHSDH, UniProtKB/Swiss-Prot accession number P0AET8.1; BSH, UniProtKB/Swiss-Prot accession number Q9KK62.1.

### Animal experiments.

Female C57 mice 8 to 10 weeks old were purchased from the Beijing Vital River Laboratory Animal Technology. All mice were kept in a specific-pathogen-free barrier facility. Mice were randomly divided into 7 groups, with 5 to 8 mice in each group. The bile acid treatment group received oral administration of 300 mg/kg body weight of UDCA and 50 mg/kg body weight of LCA dissolved in PBS; the LBP treatment groups (BAC group, *C.S* group, *B.O* group, *E.L* group, *E. L+C.S* group) were given preventive oral administration of bacterial cells as a total of ~1 × 10^9^ CFU in a 300-μL suspension. For oral gavage of multiple strains, the total CFU was 1 × 10^9^ (i.e., 3.3 × 10^8^ CFU for each strain in the BAC). The preventive oral administration was performed daily for 10 days to ensure bacterial colonization, while the control group was given an equal volume of BHI. Starting from day 11, 2% DSS was added to the drinking water to replace the normal drinking water for 7 days until the mice were sacrificed. During the whole period, oral administration of bacteria continued. Serum, liver, and colon samples were collected and stored in a freezer at −80°C.

**(i) Ethics statement.** All animal experiments were approved by the Institutional Animal Care and Use Committee at the Shenzhen Institutes of Advanced Technology (SIAT-IACUC-20201218-HCS-WSWZZX-DL-A0863-02).

### Histology.

Colon tissue for histology was collected from the middle colon (1 to 2 cm), fixed in 4% paraformaldehyde overnight, and then frozen in Tissue-Tek OCT compound (Sakura Tissue-Tek catalog number 4583). The colon sections (10 μm thick) were stained with a hematoxylin-eosin stain kit (Solarbio catalog number G1120) and then viewed with a digital slide scanner (Pannoramic Midi, 3DHISTECH). Total histology scores were assessed based on the Dieleman scoring system, which is based on the sum of the 4 subscores (inflammation severity, inflammation extent, crypt damage, and percentage involvement) (44, 45).

### Targeted metabolomics of bile acids.

For *in vitro* assays of bile acid metabolism, conjugated bile acids (TUDCA and TCDCA) or primary bile acid (CDCA) were added to a bacterial culture at a final concentration of 50 μM (for each substrate) and incubated for 60 h. A 200-μL aliquot of the bacterial culture was acidified to pH 1 by using 6 N HCl, and the culture was then extracted twice using 2 mL of ethyl acetate. The combined organic extracts were then air dried and reconstituted in 50% methanol (MeOH) in double-distilled water (ddH_2_O) for liquid chromatography-mass spectrometry (LC/MS) analysis. For mouse fecal samples, 10 mg of mice feces was mixed with magnetic beads (1-mm diameter; zirconia and silica), 180 μL solvent (methanol:water:formic acid at 74:25:1), and 20 μL internal standard d4-CDCA (25 μg/mL). The mixtures were then vortexed at 4°C for 30 min, centrifuged at 12,000 × *g* for 15 min, and then filtered through a 0.22-μm microporous membrane before LC/MS analysis. LC/MS analysis was performed using a 1290 infinity UPLC system coupled online to an Agilent Technologies 6470 quadrupole mass spectrometer via an electrospray ionization interface. A Phenomenex 2.60-μm, C_18_ 100- by 4.5-mm LC column was used to perform chromatographic separation. The mobile phase consisted of A (acetone + 0.1% formic acid):B (water + 0.1% formic acid) at a 0.3-mL/min flow rate. The gradient of the mobile phase was set as follows: 0 to 2 min, 25:75; 8 min, 70:30; 8 to 15 min, 70:30; 16 min, 25:75; 16 to 22 min, 25:75. The source parameters were set as follows: spray voltage, 3,500V; sheath gas, 45 arbitrary units; aux gas, 25 arbitrary units; sweep gas, 10 arbitrary units; ion transfer tube temperature, 300°C; vaporizer temperature, 350°C. Calibration curves included at least 10 concentrations ranging from 156 to 5,000 nM (*R*^2^ > 0.99). The data were normalized by the weight of feces.

### Luminex immunoassay.

A Luminex immunoassay was performed by Guangzhou XunYi Biotechnology Co., Ltd. Bio-Plex Pro Mouse cytokine 23-plex assays were purchased from Bio-Rad and used according to the manufacturer’s recommendations with modifications as described below. Magnetic microspheres were added to the black 96-well reaction plate and washed in a Bio-Plex wash buffer. Samples were added to the plate and washed twice with wash buffer, then incubated at room temperature for 30 min with 850 rpm shaking. The plate was washed three times in Bio-Plex wash buffer, and then the antibody was added for 30 min at room temperature with shaking. After the antibody incubation, the residual solution was removed and Streptavidin-phycoerythrin (SA-PE) was added. Next, incubation continued for 10 min at room temperature, then the plate was washed three times, and the sample was resuspended. A BioPlex 200 suspension chip system instrument was then run to detect samples.

### Gut barrier permeability assay.

The gut barrier permeability was measured on day 7 of DSS administration. On the day of the assay, mice were fasted for 3 h; then, each mouse was gavaged with 80 mg/mL FITC-dextran (molecular weight, 4 kDa) in 150 μL sterile water, with the unused FITC-dextran retained to measure the standard curve after serum collection. Three hours after oral gavage, 100 μL of blood was collected from the tail into serum collection tubes. The blood was spun at 1,000 × *g* for 10 min at room temperature. Then, the serum was diluted 1:3 in water, and 100 μL serum and the standard curve samples were added to the 96-well plates. The fluorescence signal (485-nm excitation, 528-nm emission) was measured by a microplate reader (Infinite 200 Pro), and the permeability values were calculated based on the standard curve.

### Metagenomic sequencing.

Bacterial genomic DNA was extracted from mouse feces by using a QIAamp PowerFecal DNA kit (Qiagen) according to the manufacturer’s instructions. DNA concentration and purification were checked, then the DNA was sent to Annoroad Genome Technology Co., Ltd. for library construction and sequencing. Sequencing libraries were sequenced on an Illumina HiSeq X-10 platform (150-bp paired-end reads). Raw reads were first filtered using fastp (v.0.23.1) with following parameters: -q 19 -u 50 -n 5 –detect_adapter_for_pe –dont_eval_duplication. Kraken 2 (version 2.0.9-beta) was used with default parameters to obtain the taxonomic classification of reads. Principal-coordinates analysis (PCoA) with Bray-Curtis distances was performed to reveal the dissimilarity of microbial community composition among groups. Linear discriminate analysis effect size (LEfSe) was performed (http://huttenhower.sph.harvard.edu/lefse/) to analyze changes in the relative abundance of microbial taxa between the treatment group and the control group.

### Reverse trancription-qPCR of tissue RNA.

Frozen colon tissues (~50 mg) were homogenized, and RNA was isolated using the miRNeasy kit (Qiagen). cDNA was synthesized from total RNA using an EasyScript first-strand cDNA synthesis supermix (Transgen). qPCR was performed using SYBR green on a Bio-Rad CFX Connect real-time system. The primers are provided in Table S1. β2-Microglobulin was chosen as the housekeeping gene for normalization (46), and relative fold changes in target gene expression were calculated using the comparative threshold cyce (2^−ΔΔ^*^CT^*) method.

### Statistical analysis.

An unpaired *t* test (parametric) or Mann-Whitney test (nonparametric) was performed for testing the differences between two groups. For three or more groups, ordinary one-way analysis of variance (ANOVA), two-way ANOVA (parametric), or a Kruskal-Wallis test (nonparametric) was performed, followed by the Bonferroni *post hoc* test. Statistical analysis was performed using GraphPad Prism (version 8.0.1; GraphPad Software Inc., La Jolla, CA, United States).

### Data availability.

Raw data were deposited into the NCBI Sequencing Read Archive (BioProject accession number PRJNA902665).
