# Supplementary material for: Amelioration of Colitis by a Gut Bacterial Consortium Producing Anti-Inflammatory Secondary Bile Acids
Source: Microbiol Spectr. 2023 Mar 21;11(2):e03330-22. doi: 10.1128/spectrum.03330-22 (PMC10101101; doi:10.1128/spectrum.03330-22)
Supplement: Supplemental file 1 — Supplemental material. Download spectrum.03330-22-s0001.pdf, PDF file, 0.4 MB [file spectrum.03330-22-s0001.pdf]

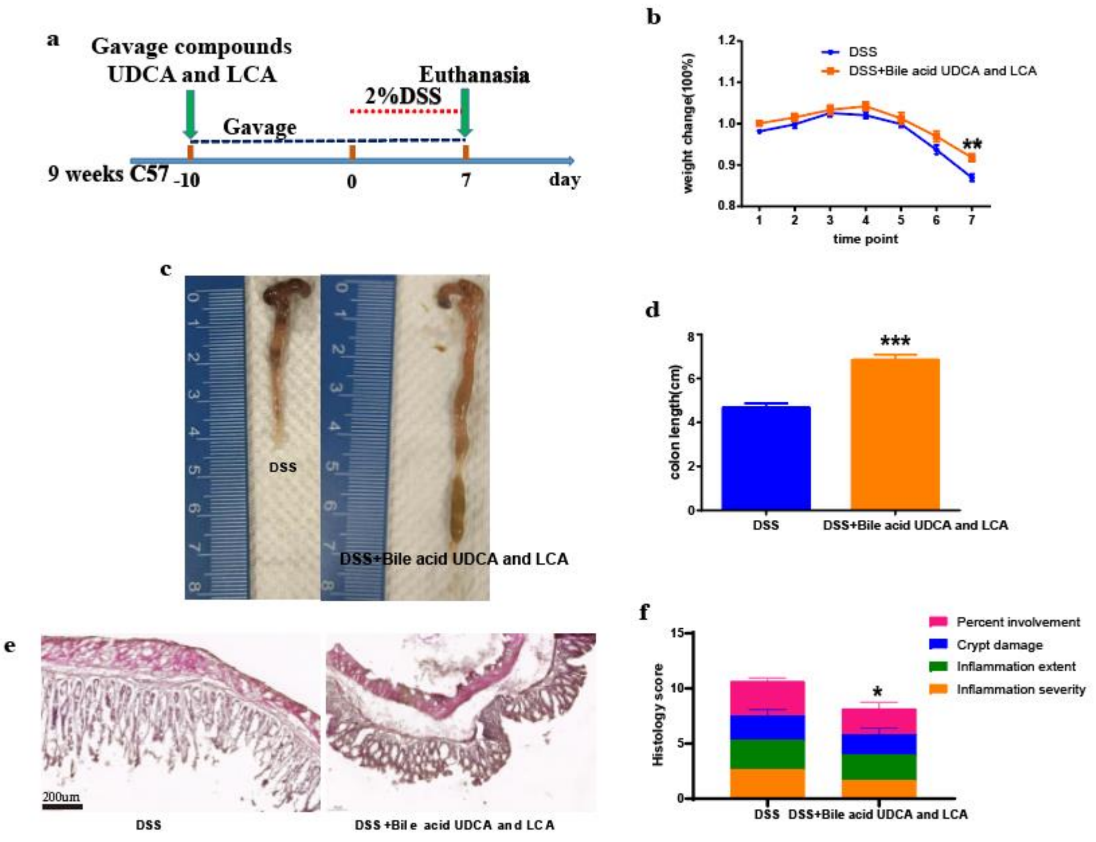

Fig S1. Bile acids LCA and UDCA alleviates the DSS-induced colitis. (a) Schematic illustration of animal model for colitis, (b) weight change. (c) Representative colon images of sacrificed mice. (d) colon length. (e-f) colon inflammation and histopathology score. Data are shown as mean ± SEM (n =7-8), Two-tailed Student's t test (or Mann-Whitney test), * $p < 0.05$, **$p < 0.01$, ***$p < 0.001$.

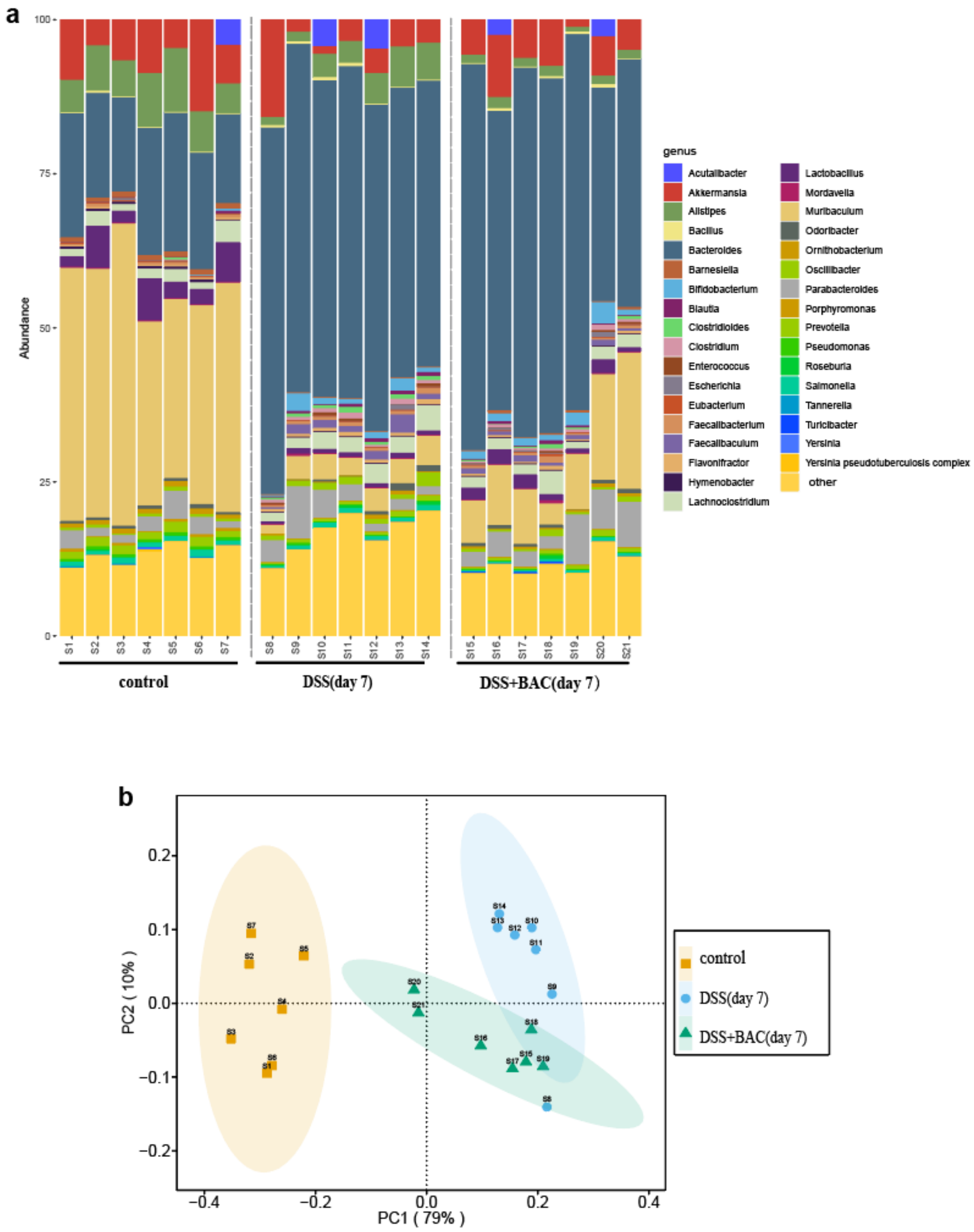

Fig S2. BAC treatment showed lessened dysbiosis in the gut microbiome caused by DSS-induced colitis. (a)Compositional profile of murine gut microbiota at the genus level. (b) PCoA of the murine gut microbiota of three different groups (n=7).

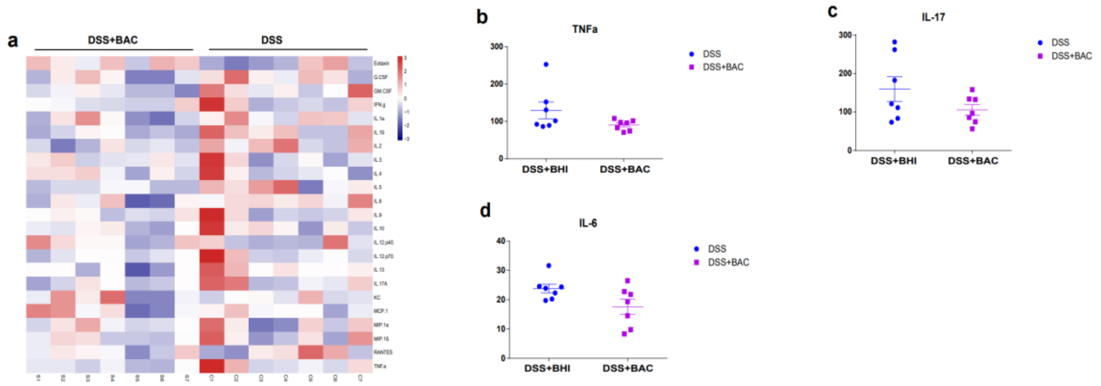

Fig S3. Luminex multiplex assays of cytokines in mice serum samples. (a, b, c, d) Luminex heatmap data show a decrease of pro-inflammation cytokines in serum. Data are shown as mean ±SEM (n=7), Two-tailed Student's t test (or Mann-Whitney test). The difference between DSS group and DSS+BAC group is not significant.

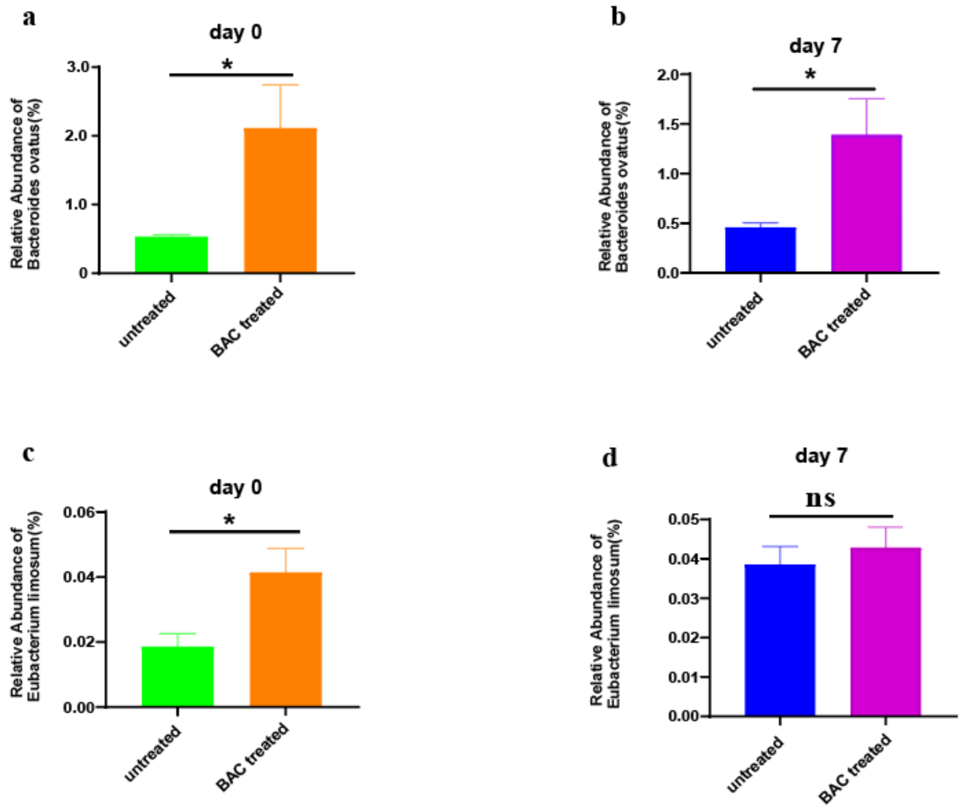

Fig S4. Relative abundance of *Bacteroides ovatus* and *Eubacterium limosum* in the untreated: mice without oral gavage of BAC and BAC treated: mice with oral gavage of BAC, started on day -10 (i.e. 10 days before DSS). (a, b) Relative abundance of *Bacteroides ovatus*. (c, d) Relative abundance of *Eubacterium limosum*. Day 0 represents the point before DSS, Day 7 represents the point of sacrifice. Data are shown as mean ± SEM (n=7), Two-tailed Student's t test (or Mann-Whitney test), * $p < 0.05$, ns is not significant.

Table S1. Primers used in qPCR assays.

Gene	Forward primer	Reverse primer
IL10	CGGGAAGACAATAACTGCACCC	CGGTTAGCAGTATGTTGTCCAGC
ZO-1	GTTGGTACGGTGCCCTGAAAGA	GCTGACAGGTAGGACAGACGAT
Claudin-1	GACAACATCGTGACCGCTCAG	TGCCAATTACCATCAAGGCTC
Claudin	GGAATATCCACCTATCACTTCAG	CATCAGCAGCAGCCATGTACTC
TGR5	GCGATGTACCCTCAACCCTG	TTGTCCCTCTTGGCTCTTCC
FXR	GCACGCTGATCAGACAGCTA	CAGGAGGGTCTGTTGGTCTG
Muc2	ATGCCCACCTCCTCAAAGAC	GTAGTTTCCGTTGGAACAGTGAA
β2m	TTCTGGTGCTTGTCTCACTGA	CAGTATGTTCGGCTTCCCATTC
